# Confirmation of infantile spasms resolution by prolonged outpatient EEGs

**DOI:** 10.1002/epi4.12540

**Published:** 2021-10-19

**Authors:** Christopher J. Yuskaitis, Kate Mysak, Brianna Godlewski, Bo Zhang, Chellamani Harini

**Affiliations:** ^1^ Division of Epilepsy and Clinical Neurophysiology Department of Neurology Boston Children's Hospital Boston Massachusetts USA; ^2^ Department of Neurology Boston Children's Hospital Boston Massachusetts USA; ^3^ Biostatistics and Research Design Center Institutional Centers for Clinical and Translational Research Harvard Medical School Boston Children's Hospital Boston Massachusetts USA

**Keywords:** 2‐week treatment response, electroencephalogram, outpatient prolonged EEG, predictive value, West syndrome

## Abstract

**Objective:**

There is no consensus on the type or duration of the posttreatment EEG needed for assessing treatment response for infantile spasms (IS). We assessed whether outpatient electroencephalograms (EEGs) are sufficient to confirm infantile spasms (IS) treatment response.

**Methods:**

Three‐year retrospective review identified new‐onset IS patients. Only presumed responder to IS treatment at 2 weeks with a prolonged (>90 minutes) outpatient EEG to assess treatment response and at least 3‐month follow‐up were included. Hypsarrhythmia, electroclinical spasms, and sleep were evaluated for the first hour and for the duration of the EEG.

**Results:**

We included 37 consecutive patients with new‐onset IS and presumed clinical response at 2 weeks posttreatment. Follow‐up outpatient prolonged EEGs (median: 150 minutes, range: 90‐240 minutes) were obtained 14 days (IQR: 13‐17) after treatment initiation. EEGs detected ongoing IS in 11 of 37 (30%) presumed early responders. Prolonged outpatient EEG had a sensitivity of 85% (confidence interval [CI] 55%‐98%) for detecting treatment failure. When hypsarrhythmia and/or electroclinical spasms were not seen, EEG had a negative predictive value 92% (CI: 75%‐99%) for confirming continued IS resolution. Outpatient EEG combined with clinical assessment, however, identified all treatment failures at 2 weeks. Compared with the entire prolonged EEG, the first‐hour recording missed IS in 45% (5/11). While sleep was captured in 95% (35/37) of the full EEG recording, the first hour of recording captured sleep in only 54% (20/37).

**Significance:**

Infantile spasms treatment response can be confirmed with a clinical history of spasm freedom and an outpatient prolonged EEG without evidence for ongoing spasms (hypsarrhythmia/electroclinical spams on EEG). Outpatient prolonged EEG, but not routine EEGs, represents an alternative to inpatient long‐term monitoring for IS posttreatment EEG follow‐up.

**Key Points**

- In our study, outpatient prolonged EEGs were greater than 90 minutes (median 150 minutes), 95% captured a full sleep cycle.
- Initial 60 minutes of EEG recordings captured sleep in only 54% of the patients and was insufficient to determine infantile spasms treatment failure.
- Prolonged EEGs without hypsarrhythmia and/or electroclinical spasms had a 92% negative predictive value to rule out ongoing IS.
- Parental reporting alone is unreliable without EEG confirmation for confirmation of initial treatment response for infantile spasms (IS).
- Outpatient prolonged EEG combined with clinical assessment, appears sufficient to identify infantile spasms treatment failures.

## 1 INTRODUCTION

Infantile spasms (IS) is an age‐specific seizure type with a high risk of future epilepsy and neurodevelopmental disorder. Prompt treatment with standard first‐line therapy, adrenocorticotropic hormone (ACTH), prednisolone, or vigabatrin (VGB) leads to improved outcomes.^1^, ^2^ Standardization of infantile spasms management has emerged as an important quality improvement metric.^3^, ^4^ Assessment of treatment response between 10 and 14 days after treatment is recommended as part of this standard of care.^3^, ^5^, ^6^

Electroencephalograms (EEGs) are essential for assessment of IS treatment response. The importance of capturing sleep on the EEG is widely accepted. However, the length of EEG monitoring at follow‐up is not standardized across institutions, including recommendations for a full 24‐hour study despite a lack of evidence.^7^ The recent COVID‐19 pandemic resulted in a shift in practice for some centers based upon consensus recommendations to utilize an outpatient EEGs with at least one sleep cycle to confirm IS resolution.^8^ The implications of these recommendations have not been studied. Specifically, it is not known whether a routine EEG length would rule out ongoing epileptic spasms.

The main goal of the study was to evaluate the ability of prolonged outpatient posttreatment EEG to predict treatment response in IS patients when assessed at 1 month and sustained response for greater than 3 months. We also evaluated whether the first hour of the study (the maximum duration of a routine EEG) was sufficient to assess treatment response.

## 2 METHOD

All patients with new‐onset IS, presumed clinical response after initial IS treatment, and a follow‐up prolonged outpatient EEG (recording duration between 90 and 240 minutes) were included as identified from our institutional EEG database between 2017 and 2020. We reserve the posttreatment EEG for patients without ongoing clinical spasms (ie, parental report of spasms resolution). Our center typically schedules the EEG to occur postfeed or at typical nap times to increase the likelihood of capturing sleep as early as possible during the EEG. Posttreatment EEG was done in those with resolution of typical clinical spasms, per parental report. Posttreatment EEG was also done in those with unclear events despite resolution of their typical spasms. Patients with persistent clinical spasms 2 weeks posttreatment are triaged to a clinical visit with escalation in therapy rather than undergoing an EEG.

Exclusion criteria included patients with EEGs not obtained for IS follow‐up and patients without at least 3‐month follow‐up data. Boston Children's Hospital institutional review board approved and waived consent for this retrospective study. Tuberous sclerosis complex (TSC) patients were not included in this study due to logistical reasons and clinical trial protocols.

We collected demographics, age at IS onset, IS treatment details, EEG reports, and clinical information. Clinical documentation for addition of IS medication, ongoing IS, and clinical relapse was assessed from IS onset to the most recent clinical encounter. Ongoing IS detected by EEG was defined as the presence of hypsarrhythmia, modified hypsarrhythmia, and/or electroclinical spasms as reported by the epileptologist's clinical EEG report. For those with electrographic resolution of IS on posttreatment EEG, follow‐up for 1‐month and 3‐month IS resolution was defined as no additional IS treatment or medication and no clinical documentation of relapse or ongoing epileptic spasms in the medical record (all notes and diagnostic reports) until at least 42 days and 3 months posttreatment, respectively.^6^ Sustained IS resolution was defined as continued response to initial treatment without relapse greater than 3 months and no documentation of additional therapy for IS at the most recent clinical follow‐up. In addition to review of the entire EEG reports, a blinded reviewer (CH) evaluated the video, raw EEG, and EMG tracings for presence of hypsarrhythmia, electroclinical spasms, or sleep from the first hour of the EEG.

Sensitivity, specificity, and positive predictive value (PPV) and negative predictive value (NPV) were calculated for the EEG to detect of ongoing IS. The primary outcome was IS response at a 1‐month clinical follow‐up after the EEG. Secondary outcomes included subsequent documentation of IS response. False negatives were defined as the EEG did not detect hypsarrhythmia/modified hypsarrhythmia and/or electroclinical spasms, but there was documentation of clinical spasms or addition of IS therapy within the month after the EEG. If clinical spasms occurred only after the 1‐month follow‐up, they were defined as an early relapse. Statistical analysis was completed using R version 3.6.2 and R studio version 1.2.5033.

## 3 RESULTS

Forty‐two patients met inclusion criteria in the prolonged EEG database of new‐onset diagnosis of IS, suspected IS resolution 2 weeks posttreatment, and at least 3‐month follow‐up. In four excluded patients, two of which were incorrectly coded as prolonged EEGs and two patients had less than 3‐month follow‐up. Thirty‐seven patients (51% male) with a median age of IS onset of 8 months (interquartile range [IQR]: 6‐12) were included in the study (Table 1). The initial diagnostic EEG noted hypsarrhythmia or modified hypsarrhythmia in 59% (22/37) of patients. All patients received standard therapy, and resolution of IS at 1‐month occurred in 65% (24/37) (Table 1). At the last follow‐up (median 411 days, IQR 163‐669), 57% (21/37) remained spasms free. No early relapses occurred between 1‐month and 3‐month follow‐up timepoints. Late IS relapse occurred in 8% (3/37). All late IS relapses occurred after 3 months posttreatment and with the timing of abrupt IS clearly documented.

**TABLE 1:** Demographics of patients with an initial posttreatment follow‐up outpatient EEG to evaluate for ongoing infantile spasms

	% (n) or median (IQR)
Male/female	
Male	51% (19)
Female	49% (18)
Age at IS onset (months)	8 (6‐12)
Initial diagnostic EEG report	
Hyps/Modified Hyps	59% (22)
No Hyps	41% (15)
Initial treatment	
ACTH	54% (20)
Prednisolone	27% (10)
Vigabatrin	19% (7)
Posttreatment EEG	
Days after treatment	14 (13‐17)
Duration of the EEG (min)	150 (120‐188) (range: 90‐240)
Hypsarrhythmia	
No	86% (32)
Yes	14% (5)
Electroclinical spasms on EEG	
No	76% (28)
Yes	24% (9)
Positive EEG^a^ for IS features	
No	70% (26)
Yes	30% (11)
Sleep captured on EEG	
No	5% (2)
Yes	95% (35)
Treatment response at 1 month and 3 months	
Responder	65% (24)
Nonresponder (ongoing IS)	35% (13)
Time to last follow‐up (days)	411 (163‐669)
Long‐term response	
Responder	57% (21)
Relapse >3 months after treatment	8% (3)
Nonresponder (ongoing IS)	35% (13)

^a^ Positive EEG = presence of hypsarrhythmia, modified hypsarrhythmia, or electroclinical spasms. Hypsarrhythmia = hypsarrhythmia and modified hypsarrhythmia.

Posttreatment EEG was obtained at a median of 14 days after treatment (IQR: 13‐17; Table 1). Median duration of the prolonged EEG was 150 minutes (IQR: 120‐188; range: 90‐240). Sleep was captured in 95% (35/37) of EEGs. The two patients without sleep on EEG had epileptiform activity on the EEG but no hypsarrhythmia or spasms, which was confirmed on a subsequent follow‐up long‐term monitoring EEG. The posttreatment EEG was normal in 8% (3/37). Epileptiform activity but not ongoing IS was identified in 62% (23/37) of EEGs. In total, the posttreatment EEG was without hypsarrhythmia and/or electroclinical spasms in 70% (26/37). Despite the parental reports of resolution of their child's typical spasms, the EEG detected ongoing IS in 30% (11/37) of the patients. The prolonged EEG captured hypsarrhythmia and electroclinical spasms on 14% (5/37) and 24% (9/37) of the studies, respectively. By definition, specificity and PPV were 100% since all patients with hypsarrhythmia or electroclinical spasms on the posttreatment EEG were defined as nonresponders and medication was added. Posttreatment EEG sensitivity for either hypsarrhythmia or electroclinical spasms to identify ongoing IS was 38% (95% confidence interval or CI: 14%‐68%) (Table 2). Posttreatment EEG sensitivity for electroclinical spasms to identify ongoing IS was 69% (CI: 39%‐91%). Combining detection of hypsarrhythmia and/or electroclinical spasms on the EEG, positive findings on the EEG increased the sensitivity and NPV to predict ongoing IS to 85% (CI: 55%‐98%) and 92% (CI: 75%‐99%), respectively. The negative likelihood ratio of ongoing IS after an outpatient prolonged EEG without hypsarrhythmia/electroclinical spasms was 0.15 (CI: 0.04‐0.55) (Table 2). For the two patients where the follow‐up EEG did not detect ongoing IS, neither had hypsarrhythmia on initial diagnostic EEG; however, follow‐up clinical notes revealed the clinicians were concerned enough by parental reports of movements and epileptiform EEG to add therapy at that time. Therefore, all patients with ongoing IS were detected by combining the results of the outpatient prolonged EEG and clinical assessment (Table 2). Selecting only patients with pretreatment hypsarrhythmia (n = 22), follow‐up EEG had persistent hypsarrhythmia in 63% (5/8) and electroclinical spasms captured in 75% (6/8) of the cases. The combination of hypsarrhythmia and/or electroclinical spasms detected 100% (8/8) of the cases of ongoing IS on the follow‐up outpatient prolonged EEG.

**TABLE 2:** Predictive value of a 2‐week posttreatment outpatient prolonged EEGs to detect ongoing infantile spasms

		Ongoing IS		Sensitivity	Specificity	PPV	NPV	NLR
		Yes	No					
		% (n)		(95% CI)	(95% CI)	(95% CI)	(95% CI)	(95% CI)
Hypsarrhythmia	Yes	13.5% (5)	0% (0)	0.38 (0.14, 0.68)	1.00 (0.86, 1.00)	1.00 (0.48, 1.00)	0.75 (0.57, 0.89)	0.62 (0.40, 0.95)
	No	21.6% (8)	64.9% (24)					
Electroclinical spasms	Yes	24.3% (9)	0% (0)	0.69 (0.39, 0.91)	1.00 (0.86, 1.00)	1.00 (0.66, 1.00)	0.86 (0.67, 0.96)	0.31 (0.14, 0.70)
	No	10.8% (4)	64.9% (24)					
Positive EEG^a^ for IS features	Yes	29.7% (11)	0% (0)	0.85 (0.55, 0.98)	1.00 (0.86, 1.00)	1.00 (0.72, 1.00)	0.92 (0.75, 0.99)	0.15 (0.04, 0.55)
	No	5.4% (2)	64.9% (24)					
Positive EEG^a^ and 2‐week clinical assessment	Yes	35.1% (13)	0%. (0)	1.00 (0.75, 1.00)	1.00 (0.86, 1.00)	1.00 (0.75, 1.00)	1.00 (0.86, 1.00)	0.00 (0.00, 0.00)
	No	0% (0)	64.9% (24)					
Positive EEG^a^ during 1st hour	Yes	21.6% (8)	0% (0)	0.62 (0.32, 0.86)	1.00 (0.86, 1.00)	1.00 (0.63, 1.00)	0.83 (0.64, 0.94)	0.38 (0.19, 0.76)
	No	13.5% (5)	64.9% (24)					

Abbreviations: EEG, electroencephalogram; IS, infantile spasms; NLR, negative likelihood ratio; NPV, negative predictive value; PPV, positive predictive value.

^a^ Positive EEG = presence of hypsarrhythmia, modified hypsarrhythmia, and/or electroclinical spasms.

We evaluated the first hour of the EEG recording to determine whether a shortened or routine EEG could be used as the posttreatment EEG for IS. Sleep was captured on 54% (20/37) of studies during the first hour of the study. Hypsarrhythmia and/or electroclinical spasms were captured on 22% (8/37) of studies during the first hour of the study. The first hour of the EEG missed 38% (5/13) of the cases of ongoing IS. The first hour of the EEG missed ongoing IS in five patients, despite documented clinical concerns for ongoing events and sleep captured within the first hour of the EEG. Four of those five patients did not have hypsarrhythmia on initial diagnostic EEG, but all five had some clinical concern for concerning events. Collectively, prediction of ongoing IS by the first hour of the EEG had a sensitivity and NPV of 62% (CI: 32%‐86%) and 83% (CI: 64%‐94%), respectively.

## 4 DISCUSSION

The results of this study demonstrate an outpatient prolonged EEG of approximately 2 hours along with clinical assessment is sufficient to detect treatment failure for IS. The outpatient EEG was sensitive to detect hypsarrhythmia or electroclinical spasms in patients with ongoing infantile spasms after initial treatment. This study is timely due to the ongoing COVID‐19 pandemic, where limiting patient exposure and reducing inpatient hospitalizations are significant concerns.

The initial 2‐week posttreatment EEG is critical to evaluate for ongoing IS, despite no studies adequately addressing the duration necessary for the evaluation.^5^ The prolonged outpatient EEG had high sensitivity to detect ongoing IS. The absence of hypsarrhythmia and/or electroclinical spasms on the EEG had a high negative predictive value for IS resolution. In the two patients where the EEG failed to identify hypsarrhythmia/electroclinical spasms, the presence of epileptiform EEG along the clinical suspicion led to escalation of therapy. Of the few studies that failed to detect ongoing IS, the majority did not have hypsarrhythmia on the initial diagnosis EEG. Although interrater reliability for evaluation of hypsarrhythmia can be low,^9^ after rereview of these EEGs, none had hypsarrhythmia or modified hypsarrhythmia. In those with pretreatment hypsarrhythmia, the follow‐up prolonged EEG confirmed IS resolution in all cases. Thus, our data support the ability of prolonged EEG to confirm IS resolution with high confidence when there is resolution of hypsarrhythmia (especially when seen on pretreatment EEG) and no captured ongoing electroclinical events. A prolonged outpatient EEG may be considered as an alternative to 24‐hour inpatient long‐term monitoring to assess IS treatment response, and provided EEG results are put into clinical context.

A routine or extended routine EEG of less than 60 minutes appears not be sensitive enough to assess treatment response in IS. We utilized the first 60 minutes of the EEG recording as a surrogate for a routine EEG. The first hour of the EEG was poor at detecting ongoing IS. It is likely due to the lack of sleep captured during the first hour of the EEG as sleep was captured only in just over half of the EEGs during the first hour. A recent report suggests they capture sleep in a 60‐minute recording in the majority of their patients^10^; however, our data do not support that view. Timing around naps, or other unknown factors, may influence the differences in our studies. Regardless, we found an EEG of at least 90 minutes (average duration of 150 minutes) increases the yield of capturing ongoing IS by 23% compared to a 60‐minute study. Routine EEGs for IS follow‐up may to falsely reassure clinicians of a treatment response potentially delaying secondary treatment.

In our cohort, 30% of the patients had ongoing IS despite the parental reports of resolution of their child's typical spasms. Hence, regardless of parental report of cessation of spasms, EEG confirmation is essential to determine IS resolution. While one may question the validity of the parental reports of ongoing spasms in our patients who did not receive EEG and went on to have the second treatment, our rigorous clinical evaluation of IS patients around day 14 by an experienced provider would eliminate that bias. Our clinical screening eliminated the need for a posttreatment EEG in patients with typical ongoing IS to avoid delays in time to second treatment, as previously reported.^10^ In a few patients, parents reported cessation of typical spasms, but after discussing with a clinician, suspicion was high enough for ongoing IS. In these cases, the patients were started on additional therapy despite the prolonged and first‐hour EEG not detecting ongoing IS. However, heavy reliance on clinical impression is potentially problematic given barriers to IS recognition even among neurologists.^11^

Limitations of our study include the potential selection bias of patients for prolonged outpatient EEGs. We did not include the patients with ongoing spasms (based on the parental report) in our study, since we do not routinely get EEGs in these patients. This limited our sample size and contributed to the width of the confidence intervals. TSC patients were not included due to logistical reasons; however, future prospective studies should include all IS etiologies. The study was conducted over a 3‐year period and is the first to rigorously evaluate outpatient EEGs and EEG duration in the context of IS treatment response. Our data provide justification for a prospective randomized trial comparing the outpatient prolonged EEG to the inpatient 24‐hour EEG in 2‐week posttreatment patients.

An outpatient prolonged EEG of at least 90 minutes with a sleep‐wake cycle along with clinical correlation appeared to be sufficient to assess electroclinical response in our patients. EEG without evidence for ongoing spasms (hypsarrhythmia/electroclinical spams on EEG) along with the clinical history of spasm freedom is reassuring.

## CONFLICT OF INTEREST

None of the authors have any conflict of interest to disclose. We confirm that we have read the Journal's position on issues involved in ethical publication and affirm that this report is consistent with those guidelines.
